# Soybean genome‑wide association study of seed weight, protein, and oil content in the southeastern USA

**DOI:** 10.1007/s00438-025-02228-8

**Published:** 2025-04-12

**Authors:** Jinesh Patel, Sejal Patel, Lauren Cook, Benjamin D Fallen, Jenny Koebernick

**Affiliations:** 1 Department of Crop, Soil and Environmental Sciences, Auburn University, Auburn, AL 36849 USA; 2 USDA, Agricultural Research Service, Raleigh, NC 27606 USA

**Keywords:** Seed oil, Protein, Seed weight, GWAS, QTL

## Abstract

Soybean is a globally significant legume crop, providing essential protein and oil for human and livestock nutrition. Improving oil and protein content simultaneously without compromising yield has been challenging due to the quantitative nature of these traits and their interrelationships. This study aims to deepen our understanding of the molecular basis soybean of seed weight, protein, and oil content to facilitate marker-assisted breeding to enhance these traits. In this research, a Genome-Wide Association Study (GWAS) was conducted utilizing 285 diverse soybean accessions from maturity group V, employing genotyping through the SoySNP50K platform. These accessions were tested in three environmental conditions of the southeast US for three traits: 100-seed weight, protein, and oil content. The study identified 18, 23, and 26 SNPs significantly associated with 100-seed weight, seed oil, and protein content. Colocalized protein and oil content regions were discovered on chromosomes 15, 16, and 20. Chromosomes 15 and 20 are well documented to have pleiotropic but opposite effects on oil and protein content, but both regions contain genes that affect individual traits, such as FAD2-1 and nodulin MtN21. A 1.92 Mb region on chromosome 11 exhibits a target region to improve oil and seed weight without affecting protein content. This study highlights key genomic regions and candidate genes influencing seed weight, protein, and oil content, with some regions affecting multiple traits. Hence, these findings provide a valuable foundation for marker-assisted selection to optimize seed weight and simultaneously enhance oil and protein content in soybean breeding programs.

## Introduction

Soybean (*Glycine max* (L.) Merrill) is a principal legume crop, serving as an important source of human food, cooking oil, and livestock feed. Soybean seeds comprise of 40% protein, 20% oil, 35% carbohydrates, and 5% minerals on a dry weight basis (Wilson 2004). The protein concentration ranges from 341 to 568 g /kg, and oil content ranges from 83 to 279 g/kg of seed weight (Wilson 2004). While soybean accessions display significant genetic diversity in seed oil and protein content, these traits are negatively correlated, and an increase in protein content often leads to a decrease in oil content (Hymowitz et al. 1972). This adverse association is mainly attributed to pleiotropy or linkage and is a major challenge in improving soybean crops (Sebolt et al. 2000; Zhang et al. 2018). In addition, seed weight is a pivotal yield component, which was modified during soybean domestication (Han et al. 2016; Duan et al. 2022), with modern cultivars weighing almost seven times more than wild accessions (Xin et al. 2016). The variation in seed weight, protein, and oil content represents complex traits that are quantitatively inherited and influenced by environmental conditions and genotype-by-environment interactions (Teng et al. 2009; Aytac and Arslanoglu 2010; Yan et al. 2017). Conventional breeding to improve and stabilize these traits simultaneously is difficult, highlighting the need to identify the genetic loci and candidate genes that enhance soybean genetic improvement (Liang et al. 2005). Understanding the molecular mechanisms and overcoming the genetic trade-offs between protein, oil, and seed weight is essential for soybean breeding to address the growing demand for these nutrients.

Several studies have been conducted using biparental population to identify QTLs for seed weight, oil, and protein content (Han et al. 2012; Kato et al. 2014; Wang et al. 2015a, 2021; Silva et al. 2021; Li et al. 2023). Over 241 QTLs associated with protein content, 316 with oil content, and 300 with seed weight have been reported across all 20 chromosomes in SoyBase, (https://soybase.org/). In 2017, Van and McHale conducted a comprehensive summary of existing research and found 175 QTLs linked to protein content, while 205 QTLs pertained to oil content. The analysis identified 55 meta-QTLs distributed across nine chromosomes, namely, Chr. 3, 5, 6, 7, 9, 14, 15, 19, and 20 (Van and McHale 2017). A meta-analysis study on SW using 65 QTLs obtained from previous studies narrowed down the information to 6 additive and 6 reductive QTLs (Qi et al. 2009). Biparental populations have been instrumental in the development of new and improved cultivars; however, inherent limitations, particularly in terms of genetic diversity and the labor-intensive process of identifying QTLs exist. The Genome-wide association study (GWAS) is a commonly used method that leverages linkage disequilibrium (LD) to pinpoint genetic variations that affect complex traits. This strategy combines genome-wide markers with phenotypic data to identify variants associated with the trait of interest (Yang et al. 2007; Sonah et al. 2015). Unlike linkage analysis, GWAS does not require the construction of a mapping population and can simultaneously analyze multiple alleles from the same locus (Yang et al. 2007). Due to the ample recombination cumulated over prolonged evolution of natural populations, GWAS yields precise resolution and sometimes even pinpoint variations within individual genes (Xu et al. 2013).

A high-throughput SNP assay, SoySNP50K, was used to genotype 18,480 G. max accessions and 1,168 G. soja accessions in the USDA Soybean Germplasm Collection (Song et al. 2015). The resulting information has been utilized to perform several GWAS studies focusing on agronomic traits, physiological, and soybean response to biotic and abiotic stress in different maturity groups (MG) of soybean (Zhang et al. 2016a; Leamy et al. 2017; Kaler et al. 2018; Lee et al. 2019; Patel et al. 2023). Hwang et al. (2014) identified 40 SNPs linked to protein content located on genomic regions across 10 chromosome and 25 SNPs associated with oil content distributed across 12 chromosomes. Sonah et al. (2015) identified 11 SNPs associated with protein and oil contents in 139 soybean lines across seven chromosomes. Yan et al. (2017) and Zhang et al. (2021) identified 17 and 11 SNPs, respectively, associated with 100-seed weight (SW) through GWAS studies. The objective of this study is to identify genomic regions and genes by GWAS that control the components of SW, oil, and protein content for MG V soybean grown in the southeastern U S. Over three years, a replicated study was conducted on 285 diverse accessions to analyze seed composition traits such as SW, oil, and protein content. The data collected from this multi-year field trial was analyzed along with SNP information from the SoySNP50K BeadChip to identify the genomic architecture of these traits through GWAS.

## Materials and methods

### Plant materials and field trials

A diversity panel of 285 MG V soybean accessions were selected based on yield, protein content, oil content, SW, disease and pest resistance, abiotic stress tolerance, lodging, and pod shattering characteristics from the USDA Germplasm Resources Information Network (GRIN) database (http://www.ars-grin.gov/). This panel was planted at the EV-Smith Field Crops Research Unit, in Shorter, AL (32.4234 ° N, 85.88816 ° W) with a CmB-Compass (loamy sand) soil type on May 6, 2020, under rainfed conditions. In 2021 and 2022 planting occurred on May 7th at the EV-Smith Plant Breeding Unit, in Tallassee, AL (32.4967 ° N, 85.8905 ° W) under irrigated conditions on a Kb-Kalmia (fine-loamy over sandy siliceous, semiactive thermic Typic Hapludult Kalmia) soil type. Plots in the 2020 season were planted in a single row in a 3-meter plot. In 2021 and 2022, the seeds were sown in two 3-meter-long rows. For all three years, replicated complete block (RCB) experimental design with two replications was employed. The plots were managed according to the Alabama Extension Soybean recommendations (Soybean IPM guide, 2020) with irrigation applied as needed in 2021 and 2022. Plots were harvested on the following dates: Oct 15, 2020, Oct 25, 2021, and Oct 19, 2022, with an Almaco R1 rotary combine, and a sub-sample was captured for post-harvest processing.

### Data collection-assessment of seed weight, oil and protein contents

Seed samples were allowed to equilibrate to room temperature for a minimum of three days to dry down prior to processing. All samples were cleaned by removing any foreign materials or off-type seed. A sub-sample of 100 seeds from each plot was used to determine a SW. Approximately 200 seeds of each plot were ground using a Perten Mill Feeder 3170. For each soybean accession, the seed protein and oil contents were measured using a Perten DA 7250 near-infrared reflectance (NIR) spectroscopy analyzer (PerkinElmer Inc., Stockholm, Sweden) and reported on a dry matter basis. Calibrations for the DA 7250 were developed and updated by the manufacturer for whole seed and ground seed samples.

### Phenotypic statistic

A statistical analysis of phenotypic data was performed in SAS (version 9.4; SAS Institute Inc., Cary, NC). Restricted maximum likelihood (REML) method was used to estimate the variance components and this estimates were used to calculate broad sense heritability following formula H^2^ = σ^2^_g_/(σ^2^_g_ + σ^2^_ge_/n + σ^2^_error_/nr), where σ^2^_g_ is the genotypic variance, σ^2^_ge_ is the genotype by environment variance, σ^2^_error_ is the residual variance, n is the number of environments, and r is the number of replications. The R package “lme4” was used to estimate the Best Linear Unbiased Prediction (BLUP) values for each trait across all environments.

### Genotypic data, population structure and linkage disequilibrium (LD) estimation

The genotyping for each accession of the population was conducted using the Illumina Infinium SoySNP50K Bead Chip (Song et al. 2013). SNPs located on unanchored sequence scaffolds or with a minor allele frequency (MAF) less than 5% in the population were excluded from the analysis. This filtering step resulted in a total of 33,453 SNPs remaining for GWAS analysis.

A set of 33,453 SNPs were used to examine the population structure of 285 accessions using STRUCTURE software Version 2.3.4 (Pritchard et al. 2000). For the analysis, the number of subsets (k) was tested ranging from 1 to 10, representing the potential number of subpopulations within the accessions. The burn-in time and Markov chain Monte Carlo iterations for each run were set to 50,000 with admixture model. Five runs were used for each stimulated value of k. The optimal number of subpopulations was determined using STRUCTURE HARVESTER (Earl and VonHoldt 2012) by estimating Delta K. A pair wise LD (r^2^) between SNPs was calculated using TASSEL 5 (Bradbury et al. 2007). LD was examined by calculating r^2^ values within a sliding window of 50 markers.

### GWAS analysis

The GWAS on SW, seed oil and protein content was performed using Fixed and Random Model Circulating Probability Unification (FarmCPU) model accounting for both principal component analysis (PCA) and kinship matrix as covariates in the Genomic Association and Prediction integrated tool version 3 (GAPIT3) (Liu et al. 2016). FarmCPU integrates fixed and random effects to account for population structure and relatedness among individuals to reduce false positive and false negative results in analysis. The significant threshold for association between marker and trait was determined using formula 1/n, where n is the number of SNPs utilized in analysis; *p* < 1/33,453 (0.000029) (or -log10(p) ≥ 4.52) whereas suggestive association threshold was set to 3.5 ≤ -log10(p) < 4.52.

### Prediction of candidate genes for seed weight, protein and oil content

Based on the LD decay distance, candidate genes were searched in the 250 kb upstream and downstream genomic regions of each peak SNP. These genes were retrieved from the reference annotation of a *Glycine max* reference genome Glyma.Wm82.a2 (Gmax2.0) from soybase (https://www.soybase.org/gb2/gbrowse/gmax2.0/, access on 01–04-2024).

## Results

### Phenotypic variations

A significant variation was found for oil, protein content, and SW across the three tested environments. The phenotypic values range from 39.8 to 52.6% for protein, 11.8–22.1% for oil content, and 6.9 g to 27.6 g for SW (Table 1). The broad-sense heritability calculations found SW, protein content, and oil content to be highly heritable, with h^2^ values of 0.86, 0.90, and 0.92, respectively (Table 1).

**Table 1:** Descriptive statistical results for seed weight, oil and protein content in soybean

Trait	Mean^c^	Max^c^	Min^c^	CV^d^	varG	VarGE	VarError	h^2^ heritability
Seed Weight ^a^	13.8	27.6	6.9	32.4	15.32	4.37	5.56	0.86
Protein Content ^b^	45.9	52.6	39.8	5.2	4.09	0.57	1.52	0.90
Oil Content ^b^	18.2	22.1	11.8	10.8	2.67	0.37	0.54	0.92

^a^ Seed weight was measured in grams

^b^ Oil and protein content were reported in percentage

^c^ Mean, Max, and Min are calculated by taking the average of the accession across multiple years

^d^ CV is coefficient of variation

### Population structure and LD decay

**Fig. 1:**
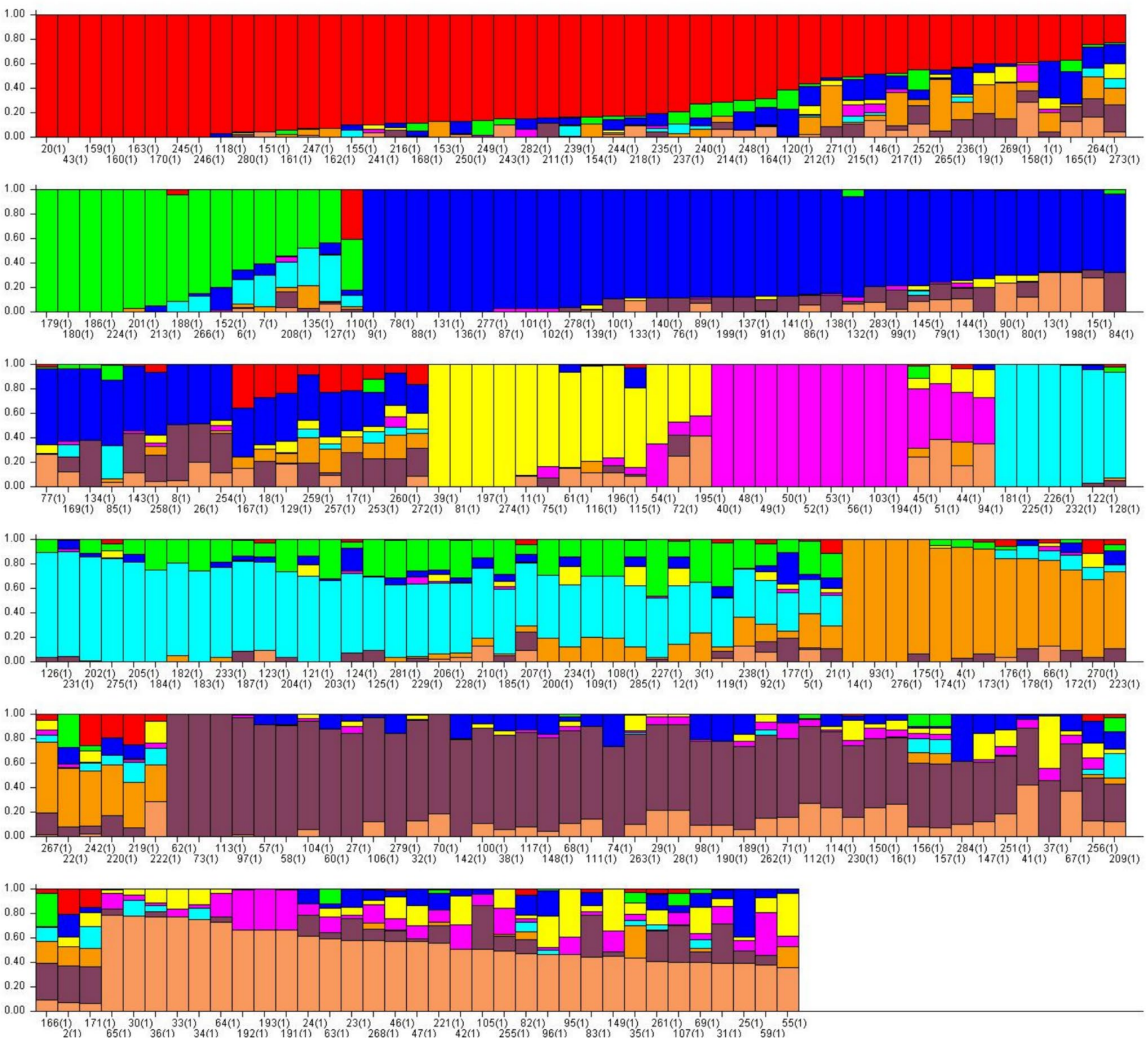
Population structure based on 33,453 SNPs distributed across 20 chromosomes. Population structure (K = 9) the areas of the different colors (red, green, blue, yellow, pink, cyan, orange, rosewood, and apricot) illustrate the proportion of each subgroup

The analysis of the population structure revealed the presence of nine subpopulations, determined through the genetic relatedness among the 285 accessions based on the distribution of 33,453 SNP loci (Fig. 1). Each accession was assigned to a specific subpopulation based on the Q value, which represented the highest level of subpopulation admixture for that accession. Most accessions in subpopulations 1, 3, and 8 originated from the United States, Japan, and Korea, respectively. Similarly, subpopulations 2, 6, and 7 were predominantly comprised of accessions from China.

**Fig. 2:**
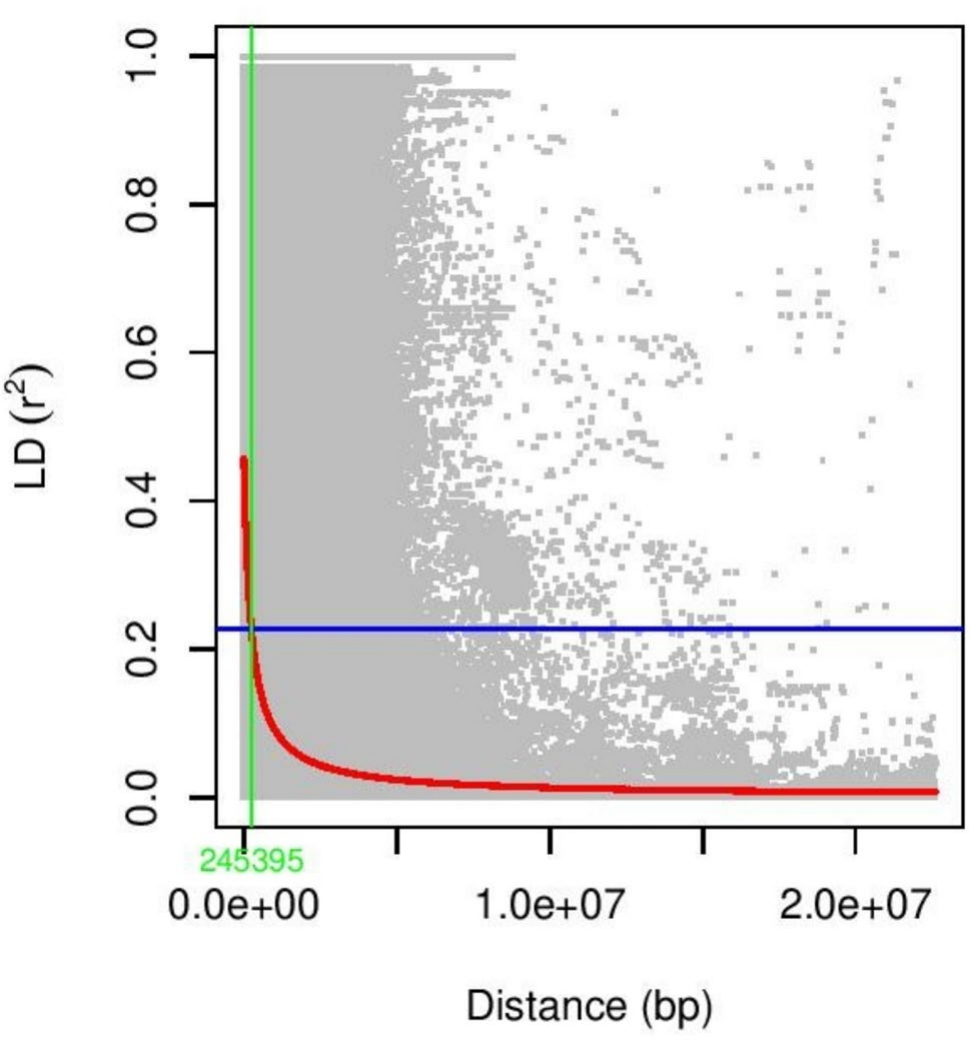
Genome-Wide Linkage Disequilibrium (LD) across 20 chromosomes. X-axis represents Linkage disequilibrium measure r^2^ and Y-axis represents the physical map distance between pairs of SNPs

Linkage disequilibrium between pairs of markers was measured in r^2^ and plotted against the physical distance. The fitting curve (red line) in Fig. 2 estimates the rate of L.D. decay. The blue line represents the half value of the power of maximum r^2^, while the green line shows the distance between two markers when the power of maximum r^2^ is halved. The estimated distance for r^2^ to drop to half from its maximum value, was about 245 kb, which aligns to previous soybean GWAS studies.

### GWAS analyses for 100-seed weight, oil, and protein content

**Fig. 3:**
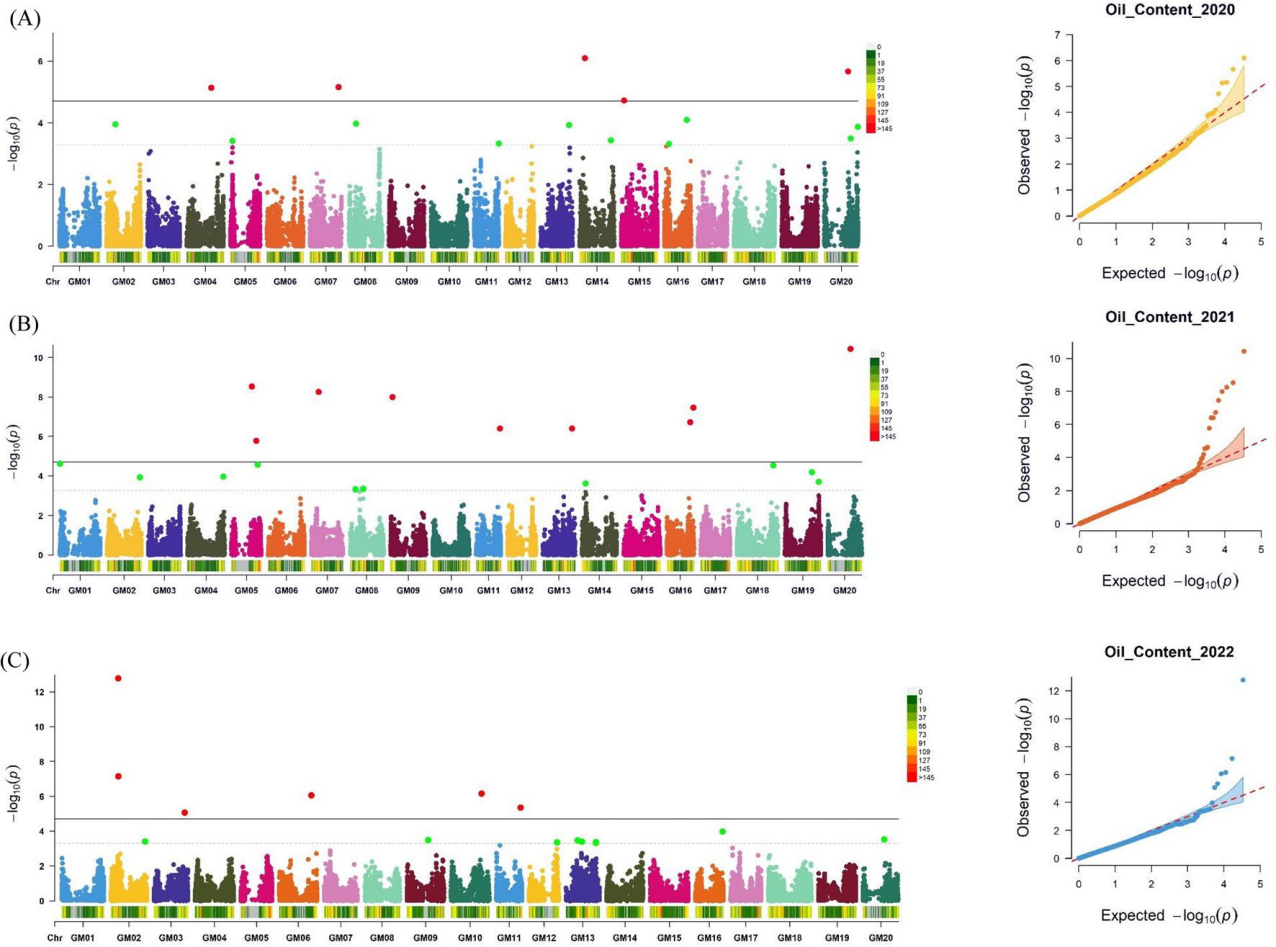
Manhattan and Q–Q plot of GWAS for oil content in a soybean association mapping panel across multiple years **A** 2020 **B** 2021 and **C** 2022. The significant associations (− logP ≥ 4.5) and suggestive associations between (3.5 ≥ − logP < 4.5) are distinguished by the threshold lines

**Fig. 4:**
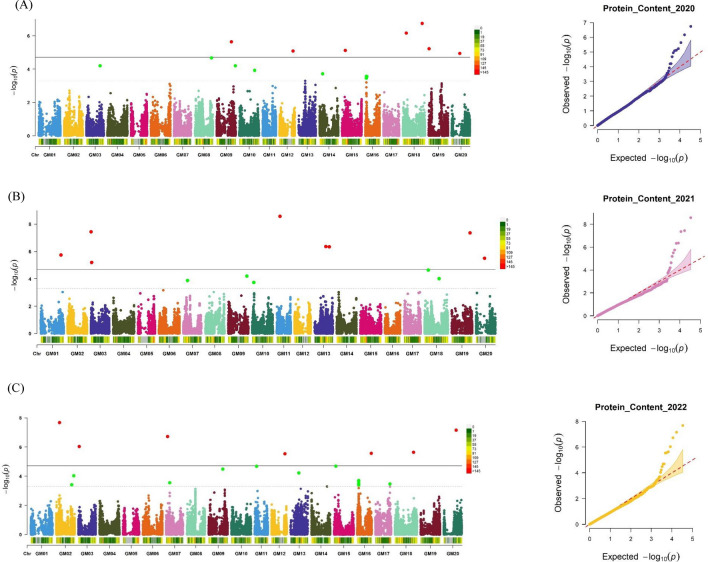
Manhattan and Q–Q plot for protein content across different years **A** 2020 **B** 2021 and **C** 2022. The significant associations (− logP ≥ 4.5) and suggestive associations between (3.5 ≥ − logP < 4.5) are distinguished by the threshold lines

**Fig. 5:**
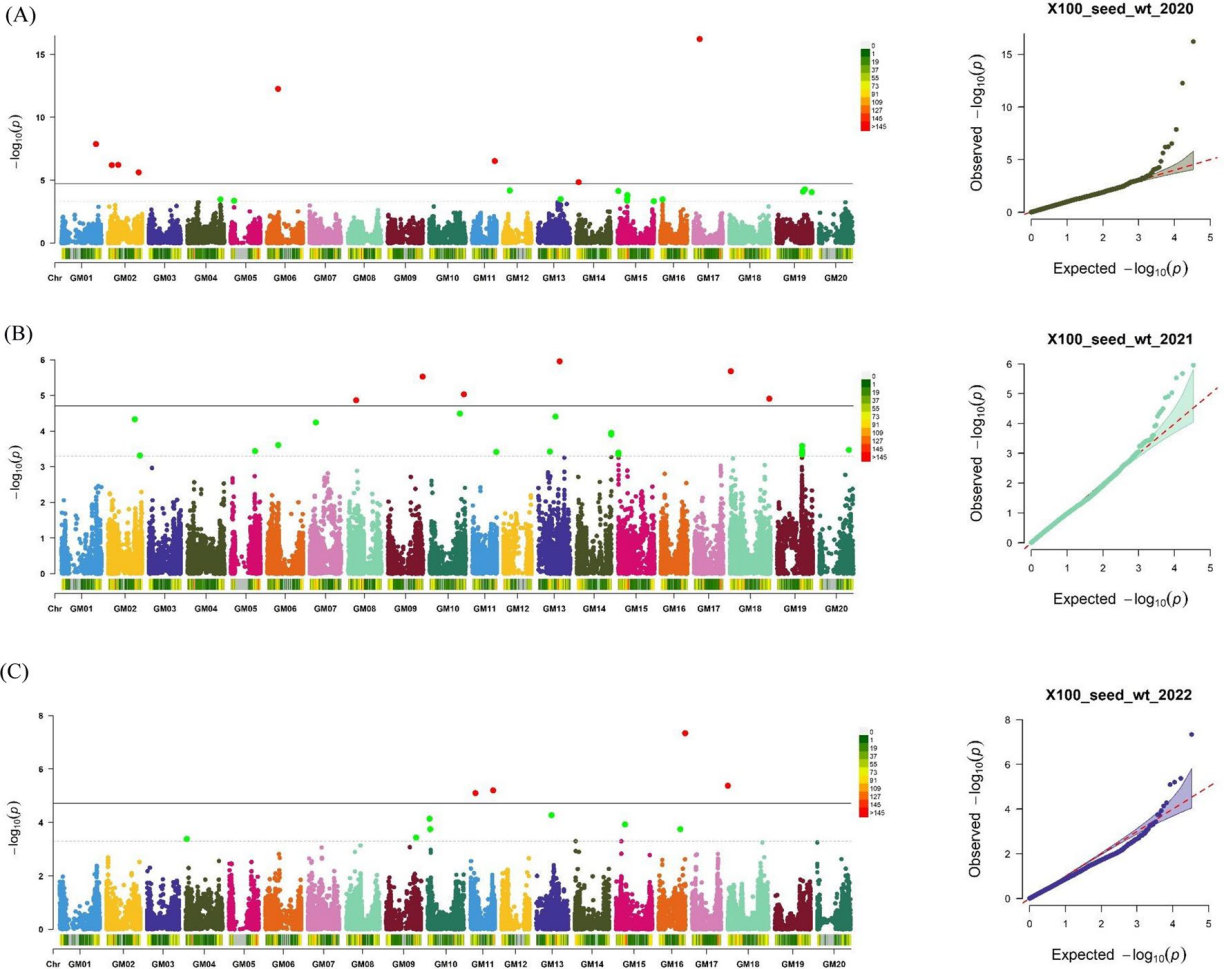
Manhattan and Q–Q plot for seed weight of 285 diverse soybean accessions over three years **A** 2020 **B** 2021 and **C** 2022. The significant associations (− logP ≥ 4.5) and suggestive associations between (3.5 ≥ − logP < 4.5) are distinguished by the threshold lines

The GWAS revealed a total of 66 significant and 49 suggestive SNPs associated with the three traits under FarmCPU model (Table 2). Five, twelve and six significant SNPs associated with oil content were respectively identified in 2020, 2021 and 2022 and one SNP marker, ss715637294 on Chr. 20 was found associated to oil content in 2021 and 2022. (Fig. 3). For protein content, eight, nine, and nine significant SNPs were detected in 2020, 2021, and 2022, respectively. Among these, one SNP marker, ss715637283 on chromosome 20, was significant in both 2020 and 2021 (Fig. 4). Eight, six, and four significant SNPs associated with SW were respectively identified in 2020, 2021 and 2022 covering twelve chromosomes. SNP marker, ss715593409 located on Chr. 06 was found significantly associated in 2020 and 2021 (Fig. 5).

**Table 2:** Allelic effects estimate of single nucleotide polymorphism (SNP) markers significantly associated (− logP ≥ 4.5) for seed oil and protein and seed weight trait in soybean

Trait	SNP	Chr ^a^	Position	*P* value	maf ^b^	effect
Oil Content-20	ss715619886	GM14	7,618,004	8.04E-07	0.308	− 0.326
Oil Content-20	ss715637354	GM20	33,722,368	2.17E-06	0.092	0.409
Oil Content-20	ss715597959	GM07	40,310,583	6.94E-06	0.127	− 0.349
Oil Content-20	ss715587515	GM04	34,775,670	7.32E-06	0.180	− 0.308
Oil Content-20	ss715621913	GM15	4,296,132	1.89E-05	0.067	0.462
Oil Content-21	ss715637294	GM20	32,282,623	3.68E-11	0.070	0.832
Oil Content-21	ss715590384	GM05	28,707,428	3.00E-09	0.252	− 0.469
Oil Content-21	ss715595750	GM07	10,283,802	5.54E-09	0.461	− 0.390
Oil Content-21	ss715603520	GM09	3,010,971	1.00E-08	0.136	0.614
Oil Content-21	ss715624938	GM16	37,086,672	3.48E-08	0.491	− 0.351
Oil Content-21	ss715624558	GM16	32,840,492	1.91E-07	0.195	− 0.388
Oil Content-21	ss715610612	GM11	33,668,616	3.98E-07	0.186	− 0.415
Oil Content-21	ss715626956	GM13	41,351,719	4.02E-07	0.224	0.390
Oil Content-21	ss715591096	GM05	35,046,834	1.70E-06	0.171	− 0.463
Oil Content-21	ss715578777	GM01	244,125	2.43E-05	0.186	− 0.329
Oil Content-21	ss715591370	GM05	36,962,030	2.73E-05	0.167	0.359
Oil Content-21	ss715631668	GM18	51,195,432	2.97E-05	0.294	0.293
Oil content-22	ss715584223	GM02	9,591,645	1.64E-13	0.426	− 0.750
Oil content-22	ss715584317	GM02	10,011,612	7.11E-08	0.477	− 0.472
Oil content-22	ss715607051	GM10	41,226,209	7.00E-07	0.308	0.401
Oil content-22	ss715594435	GM06	43,326,532	8.86E-07	0.262	0.373
Oil content-22	ss715610643	GM11	33,502,187	4.47E-06	0.443	− 0.294
Oil content-22	ss715586215	GM03	41,342,672	8.55E-06	0.498	0.323
Protein Content-20	ss715631594	GM18	50,504,231	1.82E-07	0.451	− 0.419
Protein Content-20	ss715632816	GM18	8,863,343	6.87E-07	0.257	− 0.486
Protein Content-20	ss715603764	GM09	38,923,996	2.31E-06	0.300	− 0.460
Protein Content-20	ss715633062	GM19	1,179,945	5.95E-06	0.365	0.390
Protein Content-20	ss715623168	GM15	8,829,779	7.64E-06	0.198	0.446
Protein Content-20	ss715612555	GM12	35,273,930	8.15E-06	0.310	− 0.458
Protein Content-20	ss715637283	GM20	21,083,902	1.14E-05	0.071	0.696
Protein Content-20	ss715602140	GM08	43,955,878	2.12E-05	0.463	− 0.341
Protein Content-21	ss715611141	GM11	7,763,083	2.65E-09	0.140	0.854
Protein Content-21	ss715586762	GM03	514,525	3.62E-08	0.086	− 0.853
Protein Content-21	ss715635454	GM19	45,266,984	4.38E-08	0.360	− 0.654
Protein Content-21	ss715614541	GM13	28,021,311	4.34E-07	0.114	− 0.724
Protein Content-21	ss715615803	GM13	36,686,541	4.59E-07	0.112	− 0.812
Protein Content-21	ss715579921	GM01	49,946,843	1.79E-06	0.313	0.449
Protein Content-21	ss715637283	GM20	21,083,902	3.15E-06	0.068	0.786
Protein Content-21	ss715584661	GM03	1,935,727	6.34E-06	0.259	− 0.492
Protein Content-21	ss715632866	GM18	9,154,986	2.30E-05	0.232	0.437
Protein content-22	ss715584043	GM02	8,802,340	2.09E-08	0.217	− 0.663
Protein content-22	ss715637417	GM20	34,205,370	6.97E-08	0.099	0.787
Protein content-22	ss715597749	GM07	3,858,429	1.91E-07	0.319	0.485
Protein content-22	ss715584839	GM03	2,383,419	9.27E-07	0.283	− 0.498
Protein content-22	ss715631466	GM18	49,258,868	2.32E-06	0.494	− 0.404
Protein content-22	ss715624910	GM16	36,882,604	2.72E-06	0.082	0.731
Protein content-22	ss715612558	GM12	35,302,959	2.92E-06	0.287	− 0.509
Protein content-22	ss715622862	GM15	5,848,415	2.06E-05	0.285	− 0.356
Protein content-22	ss715610809	GM11	5,011,270	2.08E-05	0.143	− 0.473
100 Seed wt-20	ss715628303	GM17	8,543,383	5.96E-17	0.425	1.401
100 Seed wt-20	ss715593409	GM06	15,781,134	5.56E-13	0.200	− 1.524
100 Seed wt-20	ss715579815	GM01	49,095,861	1.37E-08	0.298	− 1.051
100 Seed wt-20	ss715610278	GM11	32,067,520	3.01E-07	0.272	1.000
100 Seed wt-20	ss715581271	GM02	14,450,797	6.22E-07	0.262	− 0.937
100 Seed wt-20	ss715583597	GM02	5,440,843	6.40E-07	0.419	0.812
100 Seed wt-20	ss715583020	GM02	43,703,500	2.43E-06	0.440	0.738
100 Seed wt-20	ss715618230	GM14	2,967,355	1.43E-05	0.187	0.926
100 Seed wt-21	ss715614984	GM13	30,466,472	1.11E-06	0.169	− 0.964
100 Seed wt-21	ss715628872	GM18	1,340,545	2.10E-06	0.146	− 1.122
100 Seed wt-21	ss715604998	GM09	49,446,584	2.97E-06	0.496	− 0.768
100 Seed wt-21	ss715607872	GM10	48,766,650	9.36E-06	0.283	− 0.707
100 Seed wt-21	ss715632270	GM18	55,966,793	1.24E-05	0.062	1.372
100 Seed wt-21	ss715599370	GM08	12,106,889	1.36E-05	0.487	− 0.627
100 Seed wt-22	ss715625018	GM16	37,804,385	4.63E-08	0.103	1.293
100 Seed wt-22	ss715629677	GM18	200,326	4.22E-06	0.450	− 0.714
100 Seed wt-22	ss715610323	GM11	32,379,474	6.35E-06	0.433	− 0.734
100 Seed wt-22	ss715611071	GM11	7,181,102	8.02E-06	0.295	− 0.737

^a^ Chromosome

^b^ Minor allele frequency

### Gene screening

A total of 1107 soybean genes were found in the 250 kb flanking region of each peak SNP associated with all three traits; of these,135 genes lacked functional annotations. Among the annotated genes, 330, 358, and 358 were identified as potential candidates associated with oil, protein, and SW, respectively. GO enrichment analysis of these genes revealed their involvement in many pathways. For oil and protein content, these pathways included lipid binding, cellular lipid metabolic process, lipid metabolic process, lipid catabolic process, calcium-dependent phospholipid binding, phospholipid binding, protein phosphorylation, protein auto-processing, protein palmitoylation, regulation of protein export, protein catabolic process, regulation of protein stability, protein dephosphorylation, and protein glycosylation.

## Discussion

This study identifies key genomic regions and candidate genes associated with seed weight, protein, and oil content in soybean, providing valuable insights into the genetic basis of these agronomically important traits. It also highlights genomic regions that could help mitigate the negative correlation between oil and protein content. The study provides resources for marker-assisted selection and opportunities to functionally validate candidate genes and explore potential genetic improvements through gene editing or transformation techniques.

The diversity panel of 285 soybean accessions from MGV employed in this research comprises landraces collected over 50 years from diverse geographical regions, including China, Korea, Japan, the United States, Vietnam, Nepal, and Georgia. The structure analysis classifies the panel into nine distinct subpopulations. The diverse collection of accessions serves as a rich and invaluable reservoir of a wide range of allelic diversity, rendering it highly suitable for the application of GWAS. This diversity not only provides a valuable resource for advancing the field of soybean genetics but facilitates the identification of critical alleles to enhance genetic improvements in soybean.

Breeding for multiple traits is difficult, particularly when negative correlations exist, as with oil and protein content. In this study, breeding values were calculated using BLUP analysis, which helped identify a few lines that excelled in one trait while maintaining an average performance in another. For instance, FC30265 ranked among the top 20 for protein content and stood above the 50th percentile for oil content. PI437671 was placed in the top 50 for oil content and was above the 50th percentile for protein content. In total, 24 accessions were scored above the 50th percentile for both oil and protein content. These accessions look promising to develop populations that break or reduce the negative correlation between oil and protein content. It would be interesting to investigate the distribution of favorable alleles identified in this research within these lines, as well as to explore the performance of the top 10 lines in terms of oil and protein content. This could provide insight into the potential genomic regions contributing to the antagonistic nature of oil and protein.

To define a stable region conducive to integration into breeding programs, we investigated 2 MB blocks for SNPs associated with SW, oil content, and protein content in the current study and previous studies reported to soybase. Both suggestive and significant associated SNPs were searched in the 2 Mb region. However, for a region to be deemed significant, it necessitated the presence of at least one SNP showing a statistically significant association in this study. Seven genomic regions emerged as consistently associated with SW, of which three demonstrated stability across multiple environments in this study. Four genomic regions that exhibited association with SW in specific environments were also identified in other studies, confirming their relevance. Notably, one genomic region on Chr. 06 was identified in multiple environments in our study and found to be associated with SW in another study (Table 3). Similarly, this study identified 8 and 10 regions that may be pivotal zones for oil and protein content, respectively (Table 3). These regions demonstrated consistency across multiple environments or were further validated based on findings from other studies. Some of these regions warrant further evaluation, as they may contain genes affecting two or all three traits: seed weight, oil content, and protein content. For example, a 1.92 Mb region on chromosome 20 (34,205,370 − 32,282,623) contains SNPs associated with oil content in all three environments. Additionally, SNP in the region was identified as associated with protein content in 2022. This region has been reported to be a hotspot associated with protein and oil content but had the opposite effect (Vaughn et al. 2014; Lee et al. 2019; Kumar et al. 2022). Glyma.20g086900 (aldehyde dehydrogenase-related), a primary enzyme for alcohol metabolism, Glyma.20G094100 (nodulin *MtN21*) crucial for transporting amino acid to silique of Arabidopsis during early development stages, and Glyma.20g088400 (oxidoreductase, 2-oxoglutarate-Fe(II) oxygenase family protein) that is responsible for protein modification and fatty acid metabolism reside in the region (Ladwig et al. 2012; Edenberg and McClintick 2018; Zhou et al. 2021). Additionally, Glyma.20G111000 (*FAD2-1B*, omega-6 fatty acid desaturase), located 1.1 Mb away from this region, has been reported to impact oil content significantly (Kumar et al. 2022). A function assay should be designed involving these genes to understand their role in influencing oil and protein content.

**Table 3:** Identification of stable regions for seed weight, oil, and protein content within 2 mb blocks and comparing published QTLs

Trait ^a^	Year	SNP id	Chr. ^b^	Position	*p*-value	Other environment	Known QTL	References
SW	2020	ss715593409	GM06	15,781,134	5.56202E-13	2021	Seed weight 5-g7	(Zhang et al. 2016b)
SW	2022	ss715611071	GM11	7,181,102	8.02E-06		Seed weight 4-g11	(Hu et al. 2014)
SW	2020	ss715610278	GM11	32,067,520	3.01E-07	2022		
SW	2021	ss715614984	GM13	30,466,472	1.11E-06		Seed weight 6-g2	(Sonah et al. 2015)
SW	2020	ss715628303	GM17	8,543,383	5.96E-17		Seed weight 7-g8, Seed weight 12-g1	(Zhou et al. 2015; Yan et al. 2017)
SW	2021	ss715628872	GM18	1,340,545	2.10E-06	2022		
SW	2021	ss715632270	GM18	55,966,793	1.24E-05		Seed weight 5-g17	(Zhang et al. 2016b)
OC	2022	ss715586215	GM03	41,342,672	8.55E-06		Seed oil 2-g2, Seed oil 8-g1	(Zhou et al. 2015; Zhang et al. 2018)
OC	2021	ss715590384	GM05	28,707,428	2.95855E-09		Seed oil 8-g2	(Zhang et al. 2018)
OC	2021	ss715610612	GM11	33,668,616	3.98E-07	2022		
OC	2021	ss715626956	GM13	41,351,719	4.02E-07	2020	Seed oil 10-g4	(Seo et al. 2019)
OC	2020	ss715621913	GM15	4,296,132	1.89E-05		Seed oil 8-g18, Seed oil 10-g5, Seed oil 4-g11, Seed oil 11-g5	(Bandillo et al. 2015; Zhang et al. 2018; Seo et al. 2019; Yao et al. 2020)
OC	2021	ss715624558	GM16	32,840,492	1.90E-07	2020		
OC	2021	ss715624938	GM16	37,086,672	3.48E-08	2022	Seed oil 8-g21	(Zhang et al. 2018)
OC	2021	ss715637294	GM20	32,282,623	3.68E-11	2020, 2022	Seed oil 5-g8, Seed oil 6-g3, Seed oil 11-g7, Seed oil 4-g8	(Bandillo et al. 2015) (Sonah et al. 2015; Cao et al. 2017; Yao et al. 2020)
PC	2022	ss715584839	GM03	2,383,419	9.26683E-07	2021		
PC	2022	ss715597749	GM07	3,858,429	1.91E-07	2021	Seed protein 7-g18	(Zhang et al. 2017)
PC	2020	ss715602140	GM08	43,955,878	2.11871E-05		Seed protein 10-g9	(Whiting et al. 2020)
PC	2022	ss715612558	GM12	35,302,959	2.92E-06	2020	Seed protein 7-g10	(Zhang et al. 2017)
PC	2021	ss715614541	GM13	28,021,311	4.34E-07		Seed protein 3-g16	(Bandillo et al. 2015)
PC	2020	ss715623168	GM15	8,829,779	7.64E-06	2022	Seed protein 7-g13	(Zhang et al. 2017)
PC	2022	ss715624910	GM16	36,882,604	2.72E-06		Seed protein 6-g3	(Zhang et al. 2018)
PC	2020	ss715632816	GM18	8,863,343	6.87E-07	2021		
PC	2020	ss715631594	GM18	50,504,231	1.82E-07	2022		
PC	2021	ss715637283	GM20	21,083,902	3.15E-06	2020		

Another region on Chr. 15 of about 5 Mb consists of SNPs associated with oil and protein in this study and QTLs for oil and protein content have been reported in the region (Zhang et al. 2018; Lee et al. 2019; Seo et al. 2019; Yao et al. 2020). QTLs for the composition of amino and fatty acids were also discovered in this region (Zhang et al. 2018). The region contains genes involved in carbon partitioning, such as Glyma.15g049200 (sugar efflux transporter for intercellular exchange) and Glyma.15g050100 (fructose-1,6-bisphosphatase), which impact oil and protein content in soybean. It also includes three copies of genes (Glyma.15G049700, Glyma.15G049800, and Glyma.15G049900) encoding nodulin MtN21, which plays a crucial role in nitrogen allocation, loading amino acids into the root xylem, enhancing N2 fixation efficiency, and supporting seed development (Ladwig et al. 2012; Carter and Tegeder 2016; Zhang et al. 2018). Manipulating individual genes in these hotspot regions might be necessary to improve oil and protein content simultaneously.

**Fig. 6:**
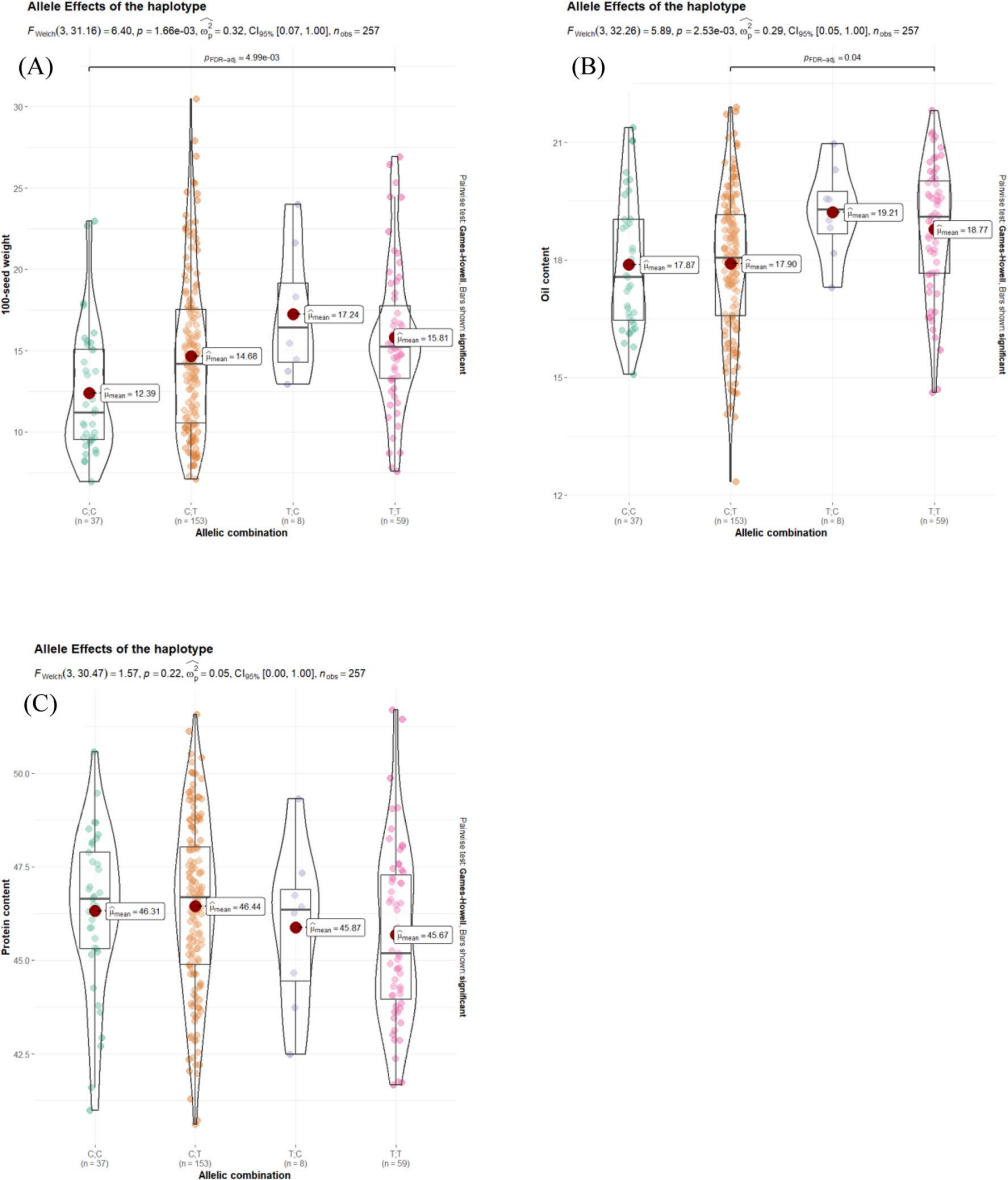
Allele effects of haplotype.distribution of allele for **A** seed weight, **B** oil content and **C** protein content of SNPs ss715610278 and ss715610612 on chromosome 11

On chromosome 11, a 1.92 Mb region spanning positions 31,753,982 to 33,668,616 has been identified, harboring SNPs associated with oil content and SW across distinct environments. This region also contains SNPs linked to linoleic, linolenic, and oleic content (Priolli et al. 2015). Evaluating the impact of significant SNPs, specifically ss715610278 for SW and ss715610612 for oil content, on seed traits reveals the potential for substantial improvement in oil content and SW without adversely affecting protein content (Fig. 6). Among the accessions, those carrying the T allele for ss715610278 and the C allele for ss715610612 exhibited promising traits. For instance, PI602493, with this allele combination, demonstrated 47.3% protein, 19.01% oil, and 17.3 g of SW. Similarly, PI408122, possessing the same allele combination, exhibited 46.2% protein, 19.56% oil, and 18.29 g of SW, values surpassing the population average. This underscores the possibility of targeting specific genomic regions associated with oil or protein traits to enhance these characteristics independently, minimizing the impact on the other trait despite the observed negative correlation between oil and protein content.

Using LD decay analysis, we searched for candidate genes around the associated SNPs. We discovered genes in the vicinity of these associated SNPs that directly influence seed weight (SW), oil content, and protein content. The GmSWEET gene family is widely recognized for its impact on seed weight, oil, and protein content in soybean (Wang et al. 2015b, 2019). Overexpression of *GmSWEET10a* or *GmSWEET10b* in soybean resulted in a significant increase in SW and a notable increase in oil accumulation but a reduction in protein levels. On the other hand, plants with knockout variant of these genes demonstrated decreased seed size and oil content but increased protein levels (Wang et al. 2020). Another gene of the same family, *GmSWEET39*, was found to improve soybean oil and protein content effectively (Zhang et al. 2020). This study identified the gene Glyma.13G264400 (*GmSWEET2*) associated with protein content, positioned near ss715615803 on Chr. 13.

A total of three (Glyma.19G011300, Glyma.19G011500, and Glyma.19G01160) copies of nodulin *MtN21* were found in the vicinity of marker ss715633062, which is associated with protein content. As mentioned, this gene family is known to impact organic nitrogen allocation and seed development. Conducting a comprehensive investigation of this gene family in soybean would be valuable for gaining insights into gene functions, evolution, and their potential roles in various global biological processes.

The keto acyl-CoA synthase (KCS) is known for affecting very long-chain fatty acid synthesis (VLCFAs). The induction of keto acyl-CoA synthase (*KCS6*) in a transgenic line increased the level of C20 and C22 in VLCFA synthesis in Arabidopsis (Hegebarth et al. 2017). This study found *KCS11* associated with oil content near ss715607051 on Chr. 10. The Mitogen-activated protein kinase (MAPK) is well known for controlling seed size and weight. The *MKK4* and *MAPK6* are positively associated with regulating grain size in rice (Duan et al. 2014; Liu et al. 2015). This study found two genes associated with SW: *MAPK3* on Chr. 8 proximate to ss715599370 and *MAPK6* on Chr. 2 near ss715581271. The cytochrome P450 family plays a vital role in many biochemical pathways, inducing flavonoids, plant metabolism, cell proliferation, and expansion (Durst and Benveniste 1993; Liu et al. 2020). In wheat plant, overexpressing cytochrome P450 gene *TaCYP78A3* resulted in increased seed size, while gene silencing reduced cell proliferation in seed coats and decreased seed size (Ma et al. 2015). In this study, two copies of cytochrome P450 genes associated with SW were identified: *CYP707A1* near ss715579815 on Chr. 1, which overlapped with three previously reported QTLs (SW 34 − 11, 36 − 17 and 37 − 5) (Han et al. 2012; Sun et al. 2012) and another cytochrome P450 gene, *CYP71B34*, on Chr. 9 near ss715604998. These genes can be further investigated to determine their relevance to the trait of interest.

## Conclusion

This study aims to identify genomic regions and genes that govern the components of SW, oil, and protein content in MGV soybean. A total of seven, eight, and ten genomic regions of less than 2 Mb were identified to have consistent associations with SW, oil, and protein content, respectively. Of these, two, two, and four are novel regions that other studies have not reported. The shared regions with previously reported QTL studies emphasize the significance of these genetic elements across different MGs and geographic regions, while unexplored regions are the outcome of the diversity panel utilized in this study or the influence of environmental factors. This GWAS study not only confirmed previous genomic regions for protein and oil contents but also revealed trait-specific loci and accessions that have the potential to mitigate the negative relationship between protein and oil contents. Further, the study identified genes like nodulin *MtN21*, *KCS*, *GMSWEET*, *MAPK*, and *CYP* that can be candidate genes for functional assay to understand their role in seed development, fatty acid, and protein biosynthesis. This data will help determine breeding strategies for improving SW, oil, and protein composition.
